# The Forms, Channels and Conditions of Regional Agricultural Carbon Emission Reduction Interaction: A Provincial Perspective in China

**DOI:** 10.3390/ijerph191710905

**Published:** 2022-09-01

**Authors:** Yanqiu He, Hongchun Wang, Rou Chen, Shiqi Hou, Dingde Xu

**Affiliations:** College of Management, Sichuan Agricultural University, Chengdu 611130, China

**Keywords:** agriculture, carbon emission, regional emission reduction interaction, group effect, technology spillover, the partitioned spatial Durbin model

## Abstract

Agricultural emission reduction is a key objective associated with sustainable agricultural development and a meaningful way to slow down global warming. Based on the comprehensive estimation of agricultural carbon emissions, this study applied the traditional spatial Durbin model (SDM) to analyze the type of regional emission reduction interaction and explore whether it is a direct or an indirect interaction caused by technology spillovers. Moreover, geographic, economic, and technical weights were used to discuss the channels of emission reduction interactions. The partitioned spatial Durbin model was applied to explore the realization conditions of regional emission reduction interactions. We found that: (1) comprehensive emission reduction interactions were identified in various regions of China, including direct and indirect interactions, in which geographic and technical channels were the major pathways for direct and indirect emission reduction interactions, respectively; (2) regions with similar economic development levels are more likely to have direct interactions, whereas regions with low technical levels are more willing to follow the high-tech regions, and the benchmarking effect is noticeable; (3) emission reduction results promoted by economic cooperation may be offset by vicious economic competition between regions, and more emission reduction intervention measures should be given to regions with high economic development levels; (4) to achieve better technological cooperation, regions must have similar technology absorption capabilities and should provide full play to the driving force of technical benchmarks.

## 1. Introduction

The increase in carbon emissions and consequent global warming threaten human survival [1]. Carbon concentration in the atmosphere is increasing at an unprecedented rate [2], leading to severe and irreversible consequences in the climate system [3,4]. Therefore, carbon reduction has gathered the focus of global attention. Agriculture is the primary source of global carbon emissions [5,6]. Studies have revealed that the worldwide food systems are responsible for more than one-third of the global carbon emissions, with approximately two-thirds of the food system emissions originating from the agricultural sector and methane from livestock production and rice cultivation accounting for approximately 35% of food system carbon emissions [7]. Furthermore, agricultural carbon emissions increase by approximately 1% annually [8,9]. Therefore, conducting sustainable production is not only a huge challenge in the agricultural sector, but also an inevitable choice for agricultural development [10].

As a leading agricultural and a major emission country [5,11], China has always considered regional cooperation a crucial way to efficiently decrease emissions. Since the joint prevention and control mechanism of air pollution was proposed in 2010, various regions have started cooperating in decreasing pollution. In 2015, it was thus proposed to rely on regional integration to attain green and low-carbon development among regions, as well as further deepen regional coordination and cooperation to decrease emissions. In 2020, the dual-carbon goal of “carbon neutrality and carbon peaking” also highlighted the establishment of a regionally coordinated carbon reduction framework to create a synergy of regional emission reductions. Driven by the national emission reduction policy, China accomplished its carbon intensity reduction target ahead of schedule and exceeded in 2018, with rapid momentum of low-carbon development. At the same time, carbon emission reduction technologies are also developing in an orderly manner. On the one hand, advances in new energy technologies and material technologies have greatly increased the proportion of clean energy represented by nuclear energy. On the other hand, negative emission technology has developed rapidly, and there are about 40 CCUS (Carbon Capture, Utilization, and Storage) demonstration projects with a capture capacity of 3 million tons per year. This study aims to demonstrate China’s experience in regionally coordinated emission reductions and provide a reference for other countries to reduce carbon emissions.

Many studies in various countries and regions have supported a significant spatial correlation of carbon emissions [12,13,14], indicating that reliance on the unilateral actions of individual regions is impossible and, thus, cooperative regionally coordinated emission reduction actions are vital [15]. Moreover, many studies have attributed regional correlations between carbon emissions to economic, technological, and policy associations [16,17], as well as similarities in the energy consumption behavior of micro-subjects and their imitation of environmental behavior [18,19]. Alternatively, regional correlations are attributable to the variation in cross-regional output resulting from the changes in the final demand [20,21]. Some studies investigated the spatial correlation of agricultural carbon emissions to determine whether total agricultural emissions, emission intensity, emission efficiency, or net emissions have spatial spillover effects. The status of the agricultural economy, production structure, technology innovation, labor force, and urbanization affect the spatial correlation of agricultural carbon emissions [22,23], suggesting that “technology spillover” can benefit more regions [24].

The spatial correlation of carbon emissions renders the interaction of regional emission reduction a crucial way to enhance the efficiency of emission reduction. Some studies examined the interaction of carbon emission reduction between countries and regions of the aspects of policy coordination, technology coordination, and enterprise coordination. Regarding policy coordination, establishing emission reduction targets beyond national and domestic regions is the basis for implementing emission reduction cooperation [25], and coordinated policies are more conducive to promoting carbon emission reduction and the development of renewable energy at lower costs than single policies [26,27]. Zhou [28] proposed establishing cross-regional environmental protection policy, thereby breaking the administrative boundary of pollution control and promoting regional coordinated emission reduction. Luqman [29] discussed about improving the implementation effect of the CDM (Clean Development Mechanism) from the standpoint of the dynamic cooperative game, reporting that the introduction of the Shapley value cost allocation scheme could improve international cooperation in carbon emission reduction. Tapia [30] claimed that the carbon trading policy plays a limited role in promoting emission reduction cooperation, and the restriction threshold for carbon trading in developing countries can increase the effect of the policy in promoting regional coordinated emission reduction [31].

Regarding technology coordination, most studies agreed that the development of critical technologies to manage global warming is important to effectively mitigate climatic hazards [32,33]. The spatial network connection of low-carbon innovative technologies provides an opportunity to build a cross-regional synergy mechanism and green innovation development [34], and cross-industry technology research and development (R&D) can effectively enhance the efficiency of carbon emission reduction [21]. Nevertheless, regional coordination of low-carbon technology innovation needs the cooperation of policies and industries [35], and technological collaboration can only improve global collaborative emissions reductions under mean or pessimistic assumptions about the development of key low-carbon technologies and when damage is severe [36].

Regarding enterprise coordination, Wang [37] and Wang [38] claimed that cooperative carbon emission reduction strategy has more advantages than the independent carbon emission reduction approach. This is because enterprises can rationally allocate emission reduction investments, further rationalizing the emission reduction structure of the supply chain. Hau [39] highlighted that external technology R&D cooperation can effectively break through the limitations of internal resources and capabilities of SMEs, thereby exerting a positive impact on carbon emission reduction and energy saving. Mao [40] reported that optimal cooperation in emission reduction can be attained by entering into a revenue-sharing agreement between manufacturers and service providers.

Generally, coordinated emission reduction between countries and domestic regions has been recognized as a crucial way to decrease emissions. In addition, studies have discussed specific methods of cooperative emission reduction from the aspects of policy coordination, technology coordination, and enterprise coordination. However, two shortcomings persist. First, although studies have examined the spatial correlation of carbon emissions, they have not deeply examined the reasons for the regional coordination of carbon emission reduction from a theoretical standpoint and, thus, cannot summarize the possible strategies in regional coordinated emission reduction. Second, previous studies only proposed the framework of coordinated emission reduction between countries or domestic regions, or investigated the cooperation mechanism from the standpoint of micro-enterprises, but did not deeply analyze the strategic choice of regional coordinated emission reduction—is it the direct interaction of emission reduction behavior or the indirect spillovers of emission reduction technologies? Besides this, these studies failed to answer the best channels and possible conditions for inter-regional emission reduction coordination; thus, these cannot provide practical suggestions for regional emission reduction coordination.

Using 2008–2018 panel data from China, this study not only discussed the interactive strategies of regional direct emission reduction (imitation or opposition) and indirect emission reduction (technology radiation and technology learning), but also examined the conditions for coordinated emission reduction from the dimensions of economy, industry, human capital, and technological R&D capabilities. The findings can provide references for effectively promoting regional cooperation in reducing emissions, attaining carbon peaking and carbon neutrality, and eventually decelerating global warming.

## 2. Theoretical Analysis

**Figure 1:**
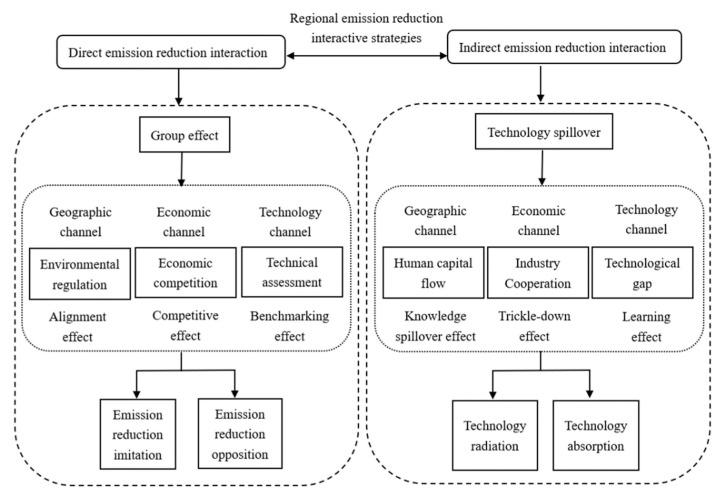
Strategic framework for regional coordinated emission reduction.

Regionally coordinated emission reduction can be performed in two ways: direct and indirect emission reduction interactions (Figure 1).

On the one hand, there is a mutual alignment of emission reduction behaviors between regions, which leads to “emission reduction imitation” or “emission reduction opposition,” that is, direct emission reduction interaction. Due to the existence of the group effect, the carbon emission decision of a region is not only affected by its own agricultural economic development, policy environment, emission reduction potential, and other factors, but also affected by the emission decision of neighboring regions. As a result, the carbon emission behavior of each region presents a certain law. The geographically neighboring regions face similar economic development policies and environmental regulatory measures. No one wants to be a “poor student” in environmental assessment, and all want to be a “good student” in economic assessment [41]. Therefore, when the emission level of a certain region decreases, the other regions also have stricter emission levels, presenting the emission reduction imitation. When a certain region relaxes its emission level and focuses on economic development, the rest of the region also relax its own emissions. There is often fierce economic competition between regions with similar economic development levels, especially regions with higher economic development levels [42]. In order to compete for or maintain their economic status, they always adjust their strategies according to the actions of their opponents. When opponents slow down their economic development and reduce emissions, the region takes this opportunity to vigorously develop its economy, thus presenting the emission reduction opposition. China takes technological innovation as its development goal, and technological development is also an important direction of emission reduction. Therefore, in order to be consistent with national goals, regions with high R&D capabilities are used as benchmarks, and other regions focus on emission reduction imitation.

Indirect emission reduction interaction is a relatively smart and continuous emission reduction interaction. Regions analyze the reasons for emission reduction in other regions and then adjust their behavior to promote emission reduction. Technology radiation and technology learning are the main indirect emission reduction interactive strategies between regions. Due to the convenient transportation, the flow cost of production factors such as human capital is lower in the neighboring regions of geographical distance, resulting in knowledge spillover. In regions with close economic relations, technology is diffused through industrial cooperation [43,44]^,^ and in regions with high economic and technological levels, its strong radiation force promotes the diffusion of resources, technology, experience, etc. to other regions, resulting in a “trickle-down effect” [45,46,47], while underdeveloped regions take the initiative to learn advanced technologies to improve carbon productivity. The existence of technological gaps between regions affects the diffusion and absorption of technology, and the effect of technology diffusion and absorption between regions with smaller technological R&D capabilities is better [48].

## 3. Materials and Methods

### 3.1. Agricultural Carbon Emission

**Figure 2:**
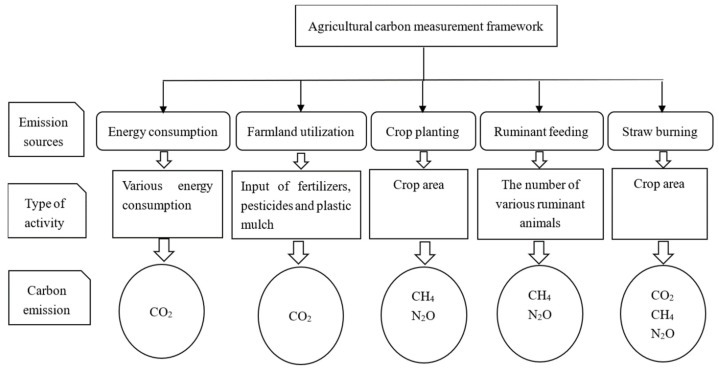
Agricultural carbon emission measurement framework.

Agricultural carbon emission sources include five categories: (1) CO_2_ produced by energy consumption; (2) CO_2_ produced by farmland utilization; (3) CH_4_ produced by growing rice and N_2_O produced from other crops; (4) CH_4_ and N_2_O produced by ruminant feeding; and (5) CO_2_, CH_4_, and N_2_O produced by straw burning. The measurement framework of agricultural carbon emission is shown in Figure 2.

The emissions of each category can be calculated as follows:

(1) $$E_{i\;}=\overset{5}{\underset{i=1}{\sum }}E_{j\;}=\overset{5}{\underset{i=1}{\sum }}(e_{j}\times f_{j})$$

where $E_{i}$ is the total emissions of a specific category; $E_{j}$ is the emissions of source *j* belonging to this category; and $e_{j}$ and $f_{j}$ represent the activity data and emission factor of source *j*, respectively. The emission factors can be found in Liu [49], Min [50], Tian [51], and Yao [52]. The GHG effects caused by 1 t of N_2_O and 1 t of CH_4_ are equivalent to those caused by 298 t of CO_2_ (81.2727 t C) and 25 t of CO_2_ (6.8182 t C), respectively [53]^,^ upon conversion into carbon equivalents. Activity data is shown in Table 1.

### 3.2. Spatial Correlation Test

Moran’s *I* was used to verify the spatial correlation of agricultural carbon emissions. The global Moran’s *I* can be calculated as follows:

(2) $$I=\frac{\sum_{i=1}^{n}\sum_{j=1}^{n}\omega_{ij}\left(y_{i}-\bar{y}\right)\left(y_{j}-\bar{y}\right)}{s^{2}\sum_{i=1}^{n}\sum_{i=1}^{n}\omega_{ij}}\;$$

(3) $$s^{2}=\frac{1}{n}\overset{n}{\underset{i=1}{\sum }}\left(y_{i}-\bar{y}\right)\;$$

(4) $$\bar{y}=\frac{\sum_{i=1}^{n}y_{i}}{n}$$

The local Moran’s *I* can be calculated as follows:

(5) $$I_{i}=z_{i}\overset{n}{\underset{j=1}{\sum }}{\bar{\omega }}_{ij}z_{j}$$

(6) $$z_{i}=\frac{y_{i}-\bar{y}}{s}$$

where I and $I_{i}$ are global and local Moran’s *I*, respectively; $y_{i}$ and $y_{j}$ are the total agricultural carbon emissions of provinces *i* and *j*, respectively; $\bar{y}$ is the average carbon emission; $\omega_{ij}$ is the element of row *i* and column *j* of the spatial weight matrix; *n* is the number of provinces; and $s^{2}$ is the variance of the total agricultural carbon emissions. According to the local agglomeration characteristics of variables, the regions were divided into four categories: high–high carbon emission agglomeration (*H–H*), low–low carbon emission agglomeration (*L–L*), high–low carbon emission agglomeration (*H–L*), and low–high carbon emission agglomeration (*L–H*).

### 3.3. Forms and Channels of Regionally Coordinated Emission Reduction—Classical SDM

To investigate the form of regionally coordinated emission reduction, the classical SDM can be used:

(7) $$\ln(AEI_{nt})=\tau_{n}\alpha +\rho \omega \ln\left(AEI_{nt}\right)+\beta_{pi}\ln\left(PI_{nt}\right)+\theta_{pi}\omega \ln\left(PI_{nt}\right)+\beta \ln\left(x_{nt}\right)+\theta \omega \ln\left(x_{nt}\right)+\mu_{n}+\upsilon_{t}+\varepsilon_{nt}\;$$

$$\varepsilon_{nt}∼N\left(0,\sigma^{2}I_{n}\right)\;$$

where $AEI_{nt}\;$represents the intensity of the agricultural carbon emissions of the 30 provinces for ten years and $PI_{nt}$ represents agricultural technology innovations. As agricultural patents directly affect agriculture [54] and patent data have strong time continuity [55], the strength of agricultural patent authorization was used to measure agricultural technological innovation. $x_{nt}\;$represents control variables, w represents spatial weight matrix, $\varepsilon_{nt}\;$represents the random error term, $\mu_{n}\;$represents individual-fixed effects, and $\upsilon_{t}\;$represents time-fixed effects. *ρ* represents the response coefficient of the emission intensity of a province to the emission intensity of other provinces and $\theta_{pi}$ represents the response coefficient of emission intensity of a province to the technology innovations of other provinces. If *ρ* is significant, it implies that there is a direct interaction between regional emission reductions. If $\theta_{pi}$ is significant, it indicates that there is an indirect interaction in regional emission reductions.

To study the realization channels of the interaction of agricultural emission reduction between regions, geographical ($w_{ijd}$), economic ($w_{ije}$), and technical difference ($w_{ijt}$) weights were selected as follows:

(8) $$\;\;\;\;\;\;w_{ijd}=\{\begin{array}{ll} -1/d_{ij}{}^{2} & \left(i\neq j\right)\\ 0 & \left(i=j\right) \end{array}$$

(9) $$w_{ije}=\{\begin{array}{ll} \frac{1}{\left|GDP_{i}-GDP_{j}\right|} & \left(i\neq j\right)\\ 0 & \left(i=j\right) \end{array}$$

(10) $$w_{ijt}=\{\begin{array}{ll} \frac{1}{\left|Tec_{i}-Tec_{j}\right|} & \left(i\neq j\right)\\ 0 & \left(i=j\right) \end{array}$$

where $d_{ij}\;$is the spherical distance between the provinces *i* and *j*; $GDP_{i}$ and $GDP_{j}$ are the agricultural added value of the provinces *i* and *j*, respectively; $Tec_{i}$ and $Tec_{j}$ are the total amount of agricultural patents granted by the provinces *i* and *j*, respectively.

Previous studies considered that the levels of agricultural economic development [56], the urbanization process [57], government environmental supervision [58], and urban–rural income gap [59] affect agricultural carbon emissions. Therefore, the variables used in the traditional SDM are defined in Table 2.

### 3.4. Condition of Regional Direct Emission Reduction Interaction—Partitioned SDM for Agricultural Carbon Emission Intensity

Facing agricultural carbon emission reduction behaviors in other regions, the responses of different regions vary [60]. Different levels of economic development in various regions prompt different agricultural economic development policies and environmental regulatory measures by the region. Therefore, local governments adopt imitation or oppositive strategies in agricultural emission reduction. Herein, the partitioned SDM for agricultural carbon emission intensity (AEI) is introduced to analyze realization conditions of the direct emission reduction interaction between regions, whether it is more likely to occur in regions with similar levels of agricultural economic development or if it can also occur in regions with significant differences in agricultural economic development levels, and also to clarify whether it is an imitation or an oppositive strategy. The weight matrix was divided according to agricultural value added per capita (*H*: regions of higher than the national average; *L*: regions of lower than the national average). The model can be provided as follows:

(11) $$\begin{array}{ll} \ln(AEI_{nt})=\tau_{n}\alpha & +[\rho_{HH}\omega_{HH}\ln\left(AEI_{nt}\right)+\rho_{LL}\omega_{LL}\ln\left(AEI_{nt}\right)+\rho_{HL}\omega_{HL}\ln\left(AEI_{nt}\right)\\ & +\rho_{LH}\omega_{LH}\ln\left(AEI_{nt}\right)]+\beta_{pi}\ln\left(PI_{nt}\right)+\theta_{pi}\omega \ln\left(PI_{nt}\right)+\beta \ln\left(x_{nt}\right)\\ & +\theta \omega \ln\left(x_{nt}\right)+\mu_{n}+\upsilon_{t}+\varepsilon_{nt}\;\; \end{array}$$

where $\rho_{HH}$*,*$\rho_{HL}$*,*$\rho_{LH}$, and $\rho_{LL}$*,* represent the interaction of emission reduction strategies among the *H–H* (high–high agricultural value added per capita agglomeration), *H–L* (high–low agricultural value added per capita agglomeration), *L–H* (low–high agricultural value added per capita), and *L–L* (low–low agricultural value added per capita) regions, respectively. The partitioned weight matrix can be expressed as follows:

(12) $$\omega =\left[\begin{array}{cc} \omega_{HH} & \omega_{HL}\\ \omega_{LH} & \omega_{LL} \end{array}\right]$$

### 3.5. Condition of Regional Indirect Emission Reduction Interaction—Partitioned SDM for Agricultural Patent Intensity (PI)

The partitioned SDM for agricultural PI was introduced to analyze the conditions for indirect emission reduction between regions. The weight matrix was divided according to the aggregation level of the agricultural industry, human resource level, and R&D level in each region (*H*: Regions where the aggregation level of the agricultural industry or the human resources level or R&D level is higher than the national average, *L*: Regions where the aggregation level of the agricultural industry or the human resources level or R&D level is lower than the national average). The model can be expressed as follows:

(13) $$\begin{array}{ll} \ln(AEI_{nt})=\tau_{n}\alpha & +\rho \omega \ln\left(AEI_{nt}\right)+\beta_{pi}\ln\left(PI_{nt}\right)+[\theta_{HH}\omega_{HH}\ln\left(PI_{nt}\right)\\ & +\theta_{LL}\omega_{LL}\ln\left(PI_{nt}\right)+\theta_{HL}\omega_{HL}\ln\left(PI_{nt}\right)+\theta_{LH}\omega_{LH}\ln\left(PI_{nt}\right)] \end{array}$$

where $\theta_{HH}\;$and $\theta_{LL}$ represent the impact of technology spillovers on emissions reductions between regions with similar levels of the agricultural industry aggregation, human resources, R&D; and$\;\theta_{LH\;}$and $\theta_{HL}$ represent the impact of technology spillovers on emissions reductions between regions with gaps in the level of the agricultural industry aggregation, human resources level, and R&D level. A negative coefficient implies that technology spillovers can benefit more regions, bringing “positive effects”. In contrast, a positive coefficient implies that technology spillovers cannot benefit other regions and cause “negative effects”.

### 3.6. Model Selection

Since this paper focuses on the interaction of regional emission reduction actions, a spatial econometric model is used. According to the general to special modeling ideas, starting from the SDM, the LR and LM tests are used to judge whether it can be simplified into the spatial lag model (SLM) and the spatial error model (SEM) [61].

From Table 3, the LR-lag and LR-error exhibit significance at 1% and 10% levels, respectively, which implies that the SDM performed better than SLM or SEM. The p-values of the LM-lag (robust) and LM-error (robust) tests are 0, indicating that the SDM could not be reduced to the SLM or the SEM. Therefore, the SDM is considered appropriate.

### 3.7. Data Sources

In this research, the primary data used, which spanned the 2008–2018 period, corresponded to 30 provinces of China. Hong Kong, Macao, Taiwan, and Tibet were excluded because of missing data. The activity data required for agricultural carbon emission estimation were obtained from the China Energy Statistics Yearbook and the China Rural Statistical Yearbook. The variables for the establishment of the SDM model were obtained from the China Rural Statistical Yearbook, the China Environmental Pollution Statistics Yearbook, and China Patent Database. In 2008, China promulgated the “2008 China Energy Conservation and Emission Reduction” report. The issue of energy conservation and emission reduction was widely publicized, and national action began. Therefore, the data began in 2008. After more than ten years of hard work, China accomplished its carbon intensity reduction target ahead of schedule and exceeded in 2018, so the study period from 2008 to 2018 is more representative for analyzing the effect of regional coordinated emission reduction.

## 4. Results

### 4.1. Analysis of Agricultural Carbon Emissions and Agricultural Technology Innovations

**Figure 3:**
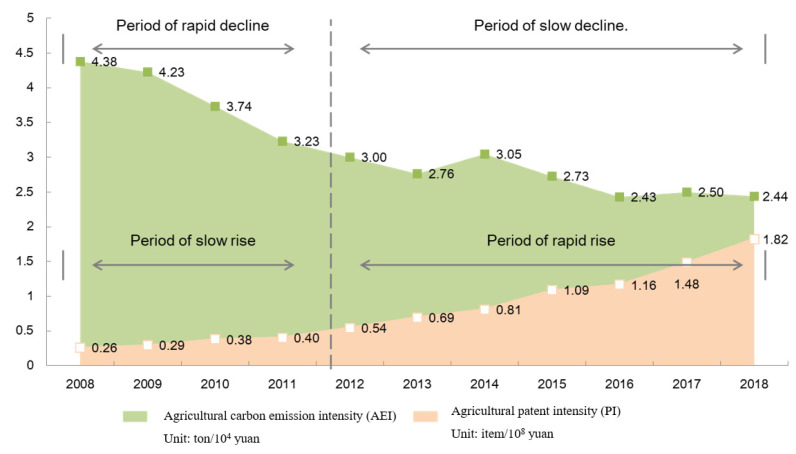
The trend of agricultural carbon emission intensity and patent intensity.

The agricultural carbon emission intensity exhibited a fluctuating downward trend, from 4.38 ton/10^4^ CNY in 2008 to 2.44 × 10^4^ ton/CNY in 2018 (Figure 3). In contrast, agricultural patent intensity exhibited an upward trend, from 0.26 items/10^8^ CNY in 2008 to 1.82 items/10^8^ CNY in 2018.

From the development stage, carbon emission intensity and patent intensity could be divided into two stages. First, during 2008–2011, the carbon emission intensity declined rapidly, and patent intensity increased slowly, with mean growth rates of −9.65% and 15.44%, respectively. During this period, under the dual influence of China’s “Eleventh Five-Year Plan” agricultural energy conservation and emission reduction targets and the commitment to reducing carbon intensity, agriculture actively enhanced the use efficiency of energy, chemical fertilizers, pesticides, and other input factors. The average annual growth rate of total carbon emissions is just 2.1%. Moreover, the proposal of the modern agricultural development strategy brought agriculture into a period of rapid development, the average annual growth rate of added value is as high as 12.8%, and the agriculture carbon intensity has declined rapidly.

In the second stage (2012–2018), carbon emission intensity declined slowly, and the patent intensity increased rapidly, with mean growth rates of −3.38% and 22.45%, respectively. During this period, China’s economic growth slowed down. Augmenting the quality of economic development and the level of agricultural science and technology became the focus of development. The growth rate of agriculture declined, and the average annual growth rate declined to 5.4%, while the total amount of agricultural carbon emissions continued to increase slowly. The contribution of scientific and technological progress in the agricultural economy has increased rapidly. The contribution rate of scientific and technological progress in the agricultural economy in 2018 increased by 6 percentage points compared with 2012. Hence, agricultural carbon intensity decreased slowly, and the agricultural patent intensity increased rapidly.

**Figure 4:**
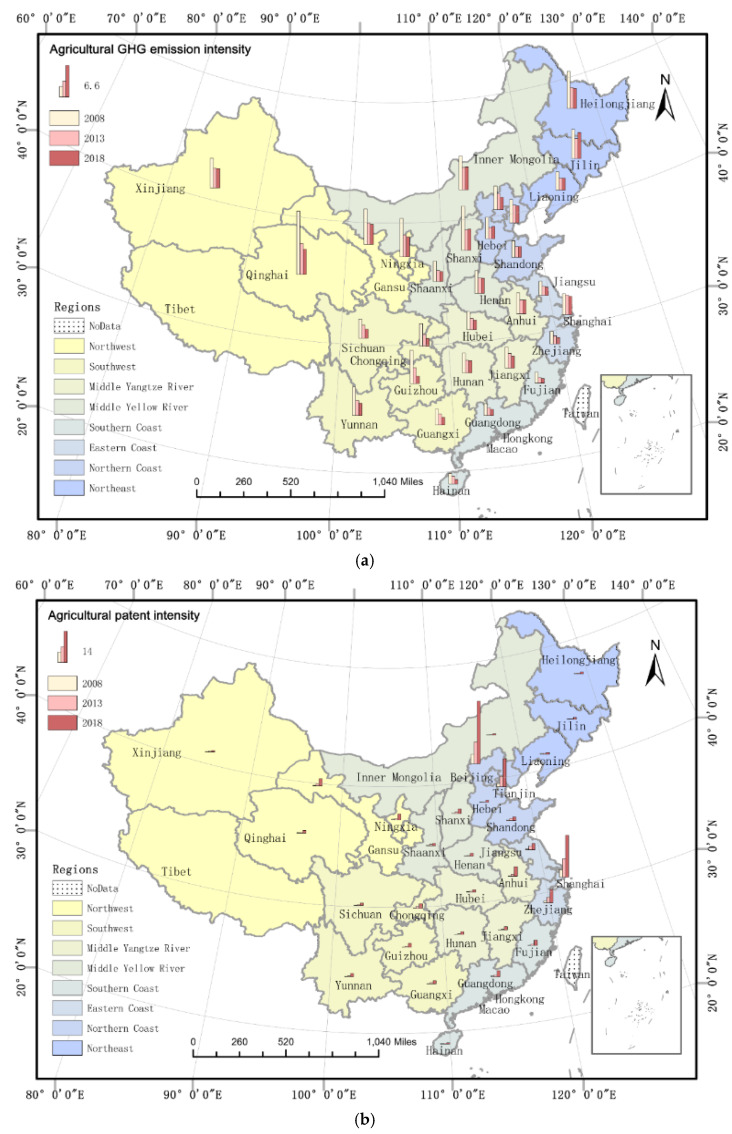
Spatial pattern of agricultural carbon emission (**a**) and patent intensity (**b**).

Figure 4a shows that the emission intensities in northeast, northwest, and middle reaches of the Yellow River were relatively high. In contrast, those of the eastern and southern coasts were relatively low, exhibiting a characteristic decreasing trend from north to south and west to east. Nonetheless, the regional differences in emission intensity gradually narrowed.

Figure 4b shows the regional differences in agricultural technology innovation levels were expanding. The technological innovation level of Beijing and Tianjin on the northern coast and Shanghai on the eastern coast was considerably higher than that of other provinces. The technological innovation exhibited a decreasing trend from south to north and from east to west. The level of agricultural technology innovation gradually improved in all provinces.

### 4.2. Spatial Correlation Test

Table 4 shows that the global Moran’s *I* index of agricultural carbon emission intensity and technology innovation were positive within 2008–2018 and significant at a 99% confidence level. These results indicate that the spatial distribution of emission intensity and technological innovation was not random but crossed regional restrictions and exhibited significant spatial agglomeration characteristics. The global Moran’s *I* index of agricultural carbon emission intensity exhibited a decreasing and then increasing trend, with a mean value of 0.259. Moreover, the global Moran’s *I* index of technological innovation was relatively stable, fluctuating from 0.260 to 0.319.

**Figure 5:**
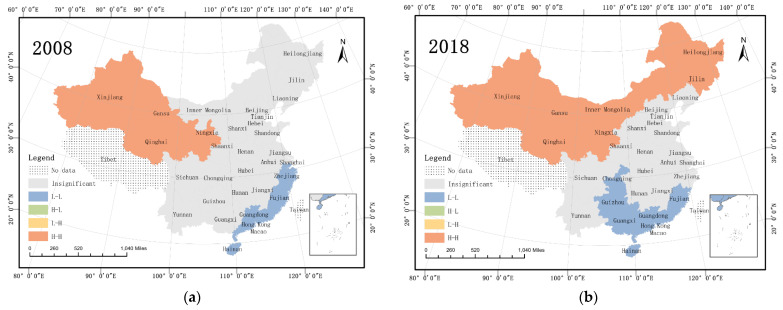

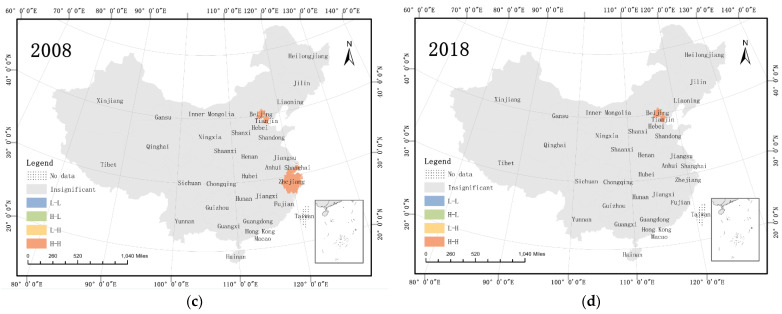
LISA cluster map of agricultural carbon emission intensity (**a**,**b**) and agricultural patent intensity (**c**,**d**).

As shown in Figure 5a,b, agricultural carbon emission intensity formed two clustering categories in the regional spatial distribution. The number of provinces with hotspot clusters (*H–H*) and coldspot clusters (*L–L*) increased, with the hotspot clusters of AEI being located in the northwest and middle reaches of the Yellow River. Some coastal provinces, such as Fujian, Guangdong, Guangxi, and Hainan, exhibited coldspot clustering.

Compared with clusters formed by agricultural carbon emission intensity, the scale of PI clusters was substantially smaller (Figure 5c,d). Comparing data from 2008 and 2018, the number of provinces with hotspot clusters decreased. In 2008, two hotspot clusters were formed, one in Beijing and Tianjin, and another in Shanghai and Zhejiang. In 2018, only one hotspot cluster remained.

### 4.3. Coordinated Emission Reduction Strategies and Channel Selection

#### 4.3.1. Choice of Regional Agricultural Coordinated Emission Reduction Strategies

To test which coordinated emission reduction strategy was active in each province, the results of SDM, SAR, SEM, and ordinary panel model (OPM) were evaluated.

Table 5 shows that the SDM, SAR, and SEM coefficients were more significant than the OPM. The spatial econometric model extracted the influence of the independent variable on the dependent variable and the spatial effect of the independent and dependent variables; therefore, the spatial model was better than the OPM. Moreover, the SDM comprehensively analyzed the interaction of emission reduction strategies between regions, exhibiting significant direct and indirect emission reduction interactions between regions. Therefore, the SDM is more suitable.

From the estimated results of the SDM, the spatial autocorrelation parameter “*ρ*” was significantly positive, indicating a direct emission reduction imitation between regions. A 1% intensity reduction (increase) in the agricultural emissions of a region led to a 0.514% intensity reduction (increase) in the emissions of the surrounding regions. Due to the institutional arrangement combining political centralization and regional economic decentralization in China [62,63], regions face similar agricultural economic development policies and environmental regulatory measures and exhibit “strategy convergence”. When the emission of a region increases and the economy develops rapidly, government officials in other regions face tremendous economic assessment pressure. Subsequently, local officials prefer economic growth to increase their chances of promotion. Conversely, when the emissions in a region decrease, government officials in other regions face greater environmental assessment pressure, which leads to the prioritization of environmental protection over economic growth [64].

Agricultural technology innovation exhibited spatial effects at *p* < 0.05 (θ*_PI_*). If the technological innovation level increased by 1%, the agricultural emission intensity of the surrounding regions were reduced by 0.096%. This shows an indirect strategic interaction between regions and that technology spillovers can benefit more regions.

#### 4.3.2. Analysis of the Interaction Channels of Regionally Coordinated Emission Reduction

Herein, geographic, economic, and technical weights were used to examine the coordinated emission reduction channels.

As shown in Table 6, the coefficients of the spatial lag term (ρ) were all significantly positive. The coefficient was the largest under geographic weight, indicating that the agricultural carbon emission reduction behavior in one region has direct strategic interaction with other regions through three channels, namely, geography, economy, and technology, exhibiting a mimic behavior of emission reduction, with geographic distance being the main channel for strategy imitation. The natural conditions and resource endowments of geographically adjacent regions had high similarities, and the “linkage effect” of carbon emission reduction was noticeable [65,66,67]. Regions with similar levels of economic development face relatively similar economic development policies and environmental regulatory measures given by the state. To become the “top students” in the development of the agricultural economy, local governments observe each other, causing economic competition and emission reduction competition to coexist [42], so that emission reduction behaviors converge. Regions with relatively small technological gaps have two-way exchanges, one-way support, and purchase services centered on technology, rendering the relationship between agricultural carbon emissions closer [41].

The spatial lag coefficients of agricultural technological innovation (θ*_PI_*) is significantly negative under the geographical and technical weights, indicating that the agricultural carbon emission reduction behavior has indirect strategic interactions with other regions through geographical and technological channels. Moreover, industrial and technology clusters in neighboring regions strengthen the geographic spillover of knowledge. The spillover costs control the scope of spillover [68], rendering technology spillover effects more likely to occur between neighboring regions. In addition, because technology spillovers are closely related to regional technology absorption capabilities [69], regions with small technological gaps have similar technology R&D capabilities and technology absorption capabilities. It is easier to achieve emission reduction interactions through technology learning. The spatial lag coefficients of agricultural technological innovation under technological weight was larger, indicating that the technological channel is the primary channel for indirect strategic interaction.

### 4.4. Analysis of Conditions for the Interaction of Emission Reduction Strategies

#### 4.4.1. Conditions for Direct Emission Reduction Strategies Interaction

**Figure 6:**
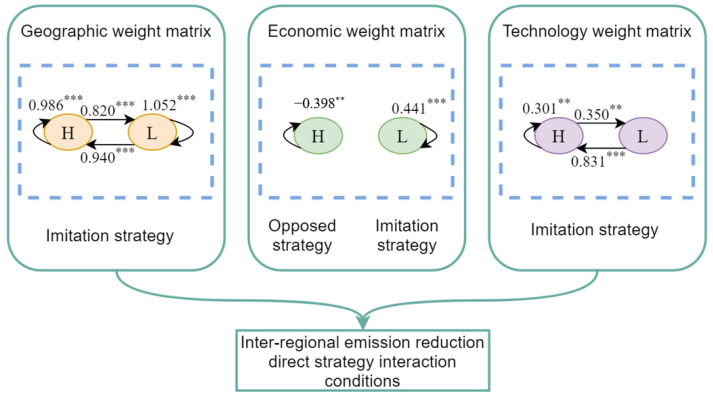
Conditions for direct emission reduction strategy interaction. Note: These coefficients are the estimated result of the partition SDM’s spatial lag term (*ρ*). ***, and ** indicate significance at the 1% and 5% levels, respectively. H: regions where per capita agricultural added value is higher than the national average, L: regions where per capita agricultural added value is lower than the national average.

As shown in Figure 6, under the geographical weight matrix, $\rho_{HH}$, $\rho_{HL}$, $\rho_{LH}$ and $\rho_{LL}$ were all positive at the 1% significance level, indicating that, no matter how the agricultural economy develops, all regions imitated the emission reduction behavior of their surrounding regions. Because regions with low agricultural economic levels face both economic and environmental pressures to prevent becoming laggards, the mimicking behavior of emission reduction among these regions was more prominent.

Under the economic weight matrix, only $\rho_{HH}$ and $\rho_{LL}$ were significant, indicating that from the perspective of economic channel, regions with similar levels of agricultural economic development were more likely to have direct emission reduction strategic interactions. $\rho_{HH}$ was significantly negative, indicating that the high economic level regions adopted opposed strategies. This is because in the competition of political performance, when one region focuses on environmental protection, other regions promote economic development, thereby relaxing environmental control [70].

Under the technology difference weight matrix, $\rho_{HH}$, $\rho_{HL}$, and $\rho_{LH}$ were all significantly positive, indicating that the imitation strategy was still the mainstay among regions under the technology channel. Furthermore, $\rho_{LH}$ was the largest, indicating that the regions with lower economic development levels had the highest degree of mimicking emission reduction to regions with higher economic development levels. The “benchmarking effect” should be fully utilized in the emission reduction interaction to drive more regional emission reductions by setting benchmark regions.

#### 4.4.2. Conditions for Indirect Emission Reduction Strategic Interaction

**Figure 7:**
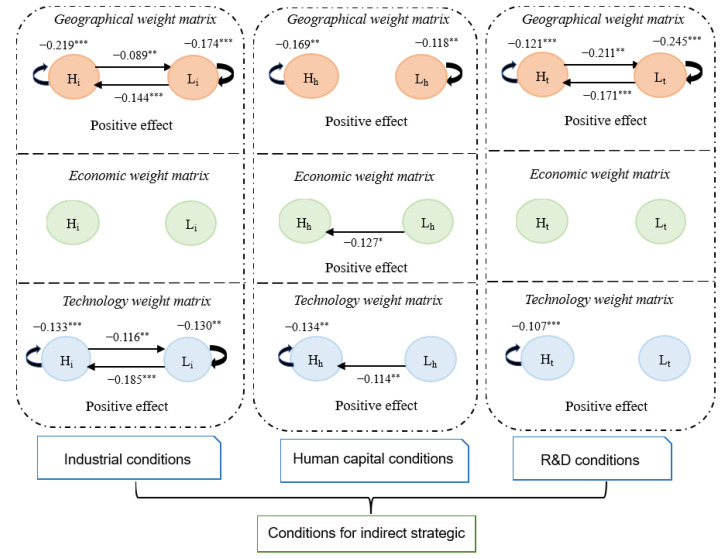
Conditions for indirect emission reduction interaction. Note: These coefficients are the estimated results of the spatial lag term (θ) of agricultural technological innovation in the partition SDM. ***, **, and * indicate significance at the 1%, 5%, and 10% levels, respectively. *Hi*: regions where the aggregation level of the agricultural industry is higher than the national average, *Li*: regions where the aggregation level of the agricultural industry is lower than the national average. *H_h_*: regions where the human resources level is higher than the national average, *L_h_*: regions where the human resources level is lower than the national average. *Ht*: regions where R&D is higher than the national average, *Lt*: regions where R&D is lower than the national average.

As shown in Figure 7, from the perspective of industrial agglomeration conditions, under the geographic and technological channels, the indirect strategy interaction occurred both in regions with similar agricultural industrial agglomeration and in regions with differences in agricultural industrial agglomeration. The effect of industrial cooperation drives technology sharing, and thus promotes more regions to achieve emission reduction. Under the geographic channel, the spillover effect of agricultural technology innovation in regions with similar industrial agglomeration levels was more prominent ($\theta_{HH}$ > $\theta_{LL}$ > $\theta_{LH}$ > $\theta_{HL}$). Among them, technology spillovers between regions with high industrial agglomeration levels had the strongest inhibitory effect on agricultural emissions due to the high agricultural industry clusters being primarily concentrated in the central and western regions of China, where agricultural production is relatively large. To achieve green development of agriculture, these regions are more proactive in reducing emissions through technological learning. Under the technology channel, the technology spillover effect of low industry agglomeration regions on high industry agglomeration regions was more robust. The effect of suppressing emissions was the greatest ($\theta_{LH}$ was the largest). The main reason is that, under the background of industrial integration, the central and western regions with a high degree of agricultural industry agglomeration have increased cooperation with the eastern regions where the agricultural industry clusters are lower by building cross-regional agricultural industry chains. Thus, through cooperation, they can incorporate advanced technology into agricultural production and improve their level of sustainable agricultural development.

From the perspective of human capital conditions, under the three channels, indirect emission reduction strategy interaction was affected by the differences in human capital levels between regions. However, indirect strategic interaction enabled more regions to achieve emission reductions (θ are all negative). Under the geographic channel, both $\theta_{HH}$ and $\theta_{LL}$ were negative at 1% significance, whereas $\theta_{HL}$ and $\theta_{LH}$ were not significant, indicating that the difference in the level of human capital in geographically adjacent regions affected the regional sharing of technical emission reduction results driven by the “knowledge spillover effect” with human resources as the carrier. Under the economic and technology channels, $\theta_{LH}$ was significant, indicating that the low human capital accumulation region produced knowledge spillovers to the high human capital accumulation region with close economic and technological relations. This is because people always seek better development opportunities and conditions. Under the “Matthew effect,” the tendency of people to move to better places is evident. Moreover, technical cooperation was also performed between regions with high human capital, resulting in a strong alliance ($\theta_{HH}$ = −0.134).

From the perspective of R&D conditions, under the geographic channel, indirect strategic interactions occurred between regions with similar R&D capabilities and regions with large gaps in R&D capabilities (the four coefficients were all significant). The coefficients are all negative, indicating that technology played an active role in reducing emissions. $\theta_{HL}$ was greater than $\theta_{LH}$, indicating that technology spillovers from regions with high R&D capabilities to regions with low R&D capabilities had a strong inhibitory effect on agricultural emissions. This is because Beijing, Tianjin, and the eastern coastal regions have provided technical assistance to many central and western regions to jointly increase agricultural productivity, reduce agricultural pollution levels, and create a phenomenon of mutual assistance between the strong and the weak. As the regions with low technological R&D capabilities are the central and western regions, where the agricultural production scale is relatively large, agricultural emissions were also higher. The emission reduction effects of mutual technology spillovers were more pronounced ($\theta_{LL}$ was greater than $\theta_{HH}$). Under the technology channel, only $\theta_{HH}$ was significantly negative, indicating that the regional technology absorption capabilities of high R&D capabilities were also relatively similar from the perspective of technological cooperation. Mutual technology spillovers were more likely to occur, thereby presenting a strong cooperation situation.

## 5. Discussion

(1) Unlike previous studies focusing on the reasons for the spatial correlation of carbon [12,20,21,23], this study analyzed and summarized the regional emission reduction interaction strategies and found two ways for the interaction of emission reduction between regions in China: (i) direct interaction of emission reduction, including imitation strategy and opposing strategy, and (ii) technical interaction. From the standpoint of direct interaction, owing to China’s relatively strict environmental assessment mechanism, to avoid administrative penalties, regions imitate each other’s carbon emission reduction behavior, but for regions with a high level of agricultural economic development, the more similar the level of economic development, the more likely it is to adopt the opposite emission reduction strategy, which differs from positive spatial correlation of carbon emissions found by some scholars [71,72,73,74]. This is because regions with a higher level of agricultural economic development have relatively fierce economic or environmental competition to compete for political performance, either choose the development idea of “economy first, environment second,” or choose the development idea of “environment first, economy second,” to take the lead in economic assessment or environmental assessment. From the viewpoint of technological interaction, scholars unanimously agreed on the existence of a technological interaction between regions [75,76,77]. Nevertheless, few studies examined the realization conditions of technological interaction. We discussed three conditions of industry, human capital, and R&D capabilities, and deduced three modes of technological interaction. First, “industrial agglomeration leads to technological interaction”. Cross-regional industrial agglomeration brings technology-sharing between regions. Geographically adjacent regions are dominated by industrial specialized agglomeration, and regions with similar technological development levels are dominated by industrial synergy agglomeration. Second, “knowledge spillovers lead to technological interaction,” which primarily occurs between regions with similar economic or technological levels, and is characterized by the cross-regional flow of human capital, but human capital does not flow from high-level regions to low-level regions. Third, the “technological R&D capability leads to technological interaction,” which is manifested as “the strong and the strong cooperating” between regions with high-tech R&D capabilities. The large gap in technological R&D capabilities affects the technology spillover between regions, and the technology threshold effect is apparent.
(2) Many studies have discussed the ways of enterprise cooperation and its impact on carbon emission reduction [78,79,80] but the improvement of enterprise cooperation awareness is inseparable from the government’s guidance [81]. Apart from this, when the region implements the coordinated joint carbon reduction model, the carbon emission reduction efforts of enterprises can also reach the peak [82], showing that the interaction of carbon emission reduction between regions can send signals to enterprises, and then promote the interaction and cooperation between regional economy, industry, and enterprises. In this study, we focused on exploring what emission reduction interaction strategies have been adopted by various regions in China under the background of regional coordinated emission reduction policies, and used geographic weight, economic weight, and technical weight to comprehensively consider whether regional emission reduction interaction is “vicious interaction” or “benign interaction”. Our findings can lay the foundation for promoting the benign interaction between enterprises in the region. For regions that implement the imitation strategy, it is crucial to guide the development of low-carbon technologies of enterprises, drive the low-carbonization of the industry, and establish a “benchmark region for emission reduction”. For regions that implement opposing strategies, it is essential to regulate the competition of enterprises, guide the benign interaction between regions, and evade the increase in carbon emissions due to vicious competition. For regions where industrial agglomeration leads to technological interaction, it is essential to promote cooperation between cross-regional enterprises, further promoting technology-sharing and transfer through economy of scale and industrial chain extension. For regions where knowledge spillovers lead to technological interaction, it is essential to guide the wider flow of human capital and promote the sharing of regional emission reduction experience. For regions where technical level leads to technical interaction, it is essential to improve the overall technical R&D ability of the region by augment the technical R&D capabilities of enterprises, thereby decreasing the problems of technical barriers to regional technical interaction.
(3) In the field of cooperative emission reduction, unlike most scholars who focused on the interaction of emission reduction between countries, we focused on the interaction of emission reduction between regions. Li [83] pointed out that Belt and Road countries can achieve economic and environmental win–win through international trade, while infrastructure investment and energy cooperation can improve energy efficiency and reduce carbon emissions by promoting advanced technologies and funds transfer [84]. Mina [85] and Shin [86] analyzed the international cooperation of REDD+ projects and found that partnerships are less likely to be created between different organization categories (across-type bridging), but tend more towards cooperation with the same types (within-type bridging). Li [87] emphasized reducing emissions through energy-related aid from high-income countries to low-income countries. Scholars all believed that cooperation is beneficial to emission reduction. Compared with regional cooperation, international cooperation obviously faces more difficulties. Therefore, regional cooperation is more important for a country to achieve emission reduction goals. By studying the emission reduction interaction between regions in China, we found that in order to stimulate emission reduction potential, it is necessary to form emission reduction benchmark regions, to drive adjacent regions to reduce emissions through the “imitation effect,” and to promote technology spillovers and technology learning. Spillover should take full advantage of industrial agglomeration and human capital flow, and technology learning should reduce technical barriers. These conclusions provide more comprehensive and feasible recommendations for inter-regional emission reduction synergies in other countries.
(4) This study discussed the coordinated strategies for low-carbon emission reduction of Chinese local governments. Currently, China’s agriculture is characterized by large-scale, industrialized, and small-scale farmers. Thus, it is not only crucial to examine the implementation path of low-carbon development from a macro-perspective but also perform comprehensive analysis from the farmers’ perspective. The better realization of regional agricultural coordinated emission reduction also warrants the cooperation of farmers. To investigate the low-carbon coordination between farmers from a micro-perspective will be the direction of future research. In addition, predicting agricultural carbon emissions under coordinated regional emission reduction, judging whether China’s carbon peaking goal can be achieved, and then guiding regions to adjust emission reduction interaction strategies, are also issues worthy of study.

## 6. Conclusions

This study analyzed the forms, channels, and conditions of China’s regional emission reduction interactions to extend China’s emission reduction experience to other countries. The conclusions are as follows: Overall, relatively comprehensive emission reduction interactions, including direct and indirect interactions caused by technology spillover, were identified in various regions of China, for which the geographic channel was the main pathway for direct emission reduction interactions and the technical channel was the main channel for indirect emission reduction interactions. The differences in economic development levels did not significantly hinder direct emission reduction interactions between regions. The differences in industrial agglomeration levels were not related to indirect emission reduction interactions between regions. In contrast, differences in human capital levels and technological R&D capabilities impacted indirect emission reduction interactions.

Finally, the following suggestions are made: (i) Improve the top-level design of emission reduction policies, establish a regional coordinated emission reduction mechanism, and augment emission reduction cooperation. Relying on the coordinated development strategy, enhance the balance of agricultural economic development among regions and prevent the adverse impact of vicious economic competition on carbon emission reduction. In addition, advocate the “rich neighbor” strategy, break down barriers to regional cooperation in emission reduction, and share experience in energy conservation and emission reduction through technical cooperation or financial cooperation. (ii) Establish benchmark regions and take full advantage of the industrial integration strategy to promote technology absorption to its radiation effect on carbon emission reduction. In addition, establish an “economic benchmark” and promote the horizontal integration or vertical integration of industries between benchmark regions and other regions, and then share emission reduction experience and technologies. Besides these, establish “emission reduction benchmark” and use the government’s environmental assessment system to guide regions to learn from emission reduction benchmark, thereby stimulating the emission reduction potential of more regions. (iii) Create an excellent technology R&D environment to promote regional technology spillover and absorption. Upgrade the intellectual property protection system, encourage enterprises, universities, and other scientific research entities to carry out technology R&D through preferential policies, such as tax relief, financial subsidies, and financial discounts, and integrate talents, capital, information, and other resources to hasten the promotion and application of technology. Furthermore, regions with low-technology R&D capabilities should increase investment in technology-intensive industries, and make full use of the industrial integration strategy to promote technology absorption, thereby driving emission reductions. (iv) Guide the flow of agricultural technical talents and exerts the “knowledge spillover” effect. Increase government guidance, improve the rate of return of production factors in regions with low human capital through preferential policies, such as taxation, to attract technical talents to flow to regions with low human capital through the “Retain talent through preferential policies” method. Finally, establish a long-term mechanism for talent flow, build a career platform, illustrate the development potential of the region, and attract technical talents to flow to regions with low human capital through the “Retain talent through career development” method.

## Figures and Tables

**Table 1:** Activity data description.

Category	Indicator	Source
Energy consumption	Amount of coal, coke, crude oil, gasoline, kerosene, diesel oil, fuel oil, electric power, and natural gas used in agricultural production	China Energy Statistics Yearbook
Farmland utilization	Application amount of fertilizers, pesticides, and agricultural film, plowing area	China Rural Statistical Yearbook
Crop planting	Planting area of rice, wheat, corn, soybeans, and vegetable	China Rural Statistical Yearbook
Ruminant feeding	Annual average stock of cattle, horses, donkeys, mules, pigs, goats, and sheep	China Rural Statistical Yearbook
Straw burning	Yield of rice, wheat, corn, soybeans, cotton, and canola	China Rural Statistical Yearbook

**Table 2:** SDM variables used in the study.

Variables	Notation	Calculation	Data Sources
Agricultural carbon emission intensity	*AEI*	Ratio of agricultural carbon emissions to agricultural added value	Section 3.1
Agricultural patent intensity	*PI*	Ratio of number of agricultural patents to agricultural added value	China Patent Database
Agricultural economy	*AGDP*	Ratio of agricultural added value to rural population	China Rural Statistical Yearbook
Urbanization ratio	*UR*	Ratio of urban population to rural population	China Rural Statistical Yearbook
Urban-rural income gap	*UIG*	Ratio of disposable income of urban residents to rural residents	China Rural Statistical Yearbook
The intensity of investment in environmental governance	*GER*	Ratio of expenditure on environmental protection to agricultural added value	China Environmental Pollution Statistics Yearbook

**Table 3:** Results of spatial panel econometric model test.

Test	Statistics	*p*-Value
LR-lag	20.17 ***	0.0052
LR-error	12.18 *	0.0948
LM-lag (Robust)	32.58 ***	0.0000
LM-error (Robust)	101.61 ***	0.0000

Note: *** and * indicate significance at the 1% and 10% levels, respectively.

**Table 4:** Global Moran’s I index.

Year	Agricultural Carbon Emission Intensity		Agricultural Patent Intensity	
	Moran’s *I*	z-Value	Moran’s *I*	z-Value
2008	0.312 ***	3.771	0.309 ***	3.857
2009	0.302 ***	3.681	0.274 ***	3.509
2010	0.272 ***	3.336	0.283 ***	3.600
2011	0.275 ***	3.355	0.315 ***	3.949
2012	0.253 ***	3.111	0.306 ***	3.818
2013	0.217 ***	2.730	0.319 ***	3.939
2014	0.178 **	2.304	0.304 ***	3.740
2015	0.147 **	1.964	0.292 ***	3.609
2016	0.271 ***	3.297	0.286 ***	3.536
2017	0.316 ***	3.767	0.264 ***	3.310
2018	0.313 ***	3.733	0.260 ***	3.959

Note: ***, and ** indicate significance at the 1% and 5% levels, respectively.

**Table 5:** Estimation results of the OPM, SEM, SAR, and SDM.

Variables	Coefficient	OPM	SEM	SAR	SDM
ln(PI)	*β_PI_*	0.00002 (−0.00)	0.005 (0.36)	0.011 (0.79)	0.008 (0.59)
ln(AGDP)	*β_AGDP_*	0.853 *** (−7.96)	−0.847 *** (−17.04)	−0.865 *** (−17.98)	−0.854 *** (−17.46)
ln(UR)	*β_UR_*	−0.390 (−1.25)	−0.244 * (−1.69)	−0.270 * (−1.83)	−0.081 (−0.52)
ln(GER)	*β_GER_*	0.130 *** (−3.37)	−0.134 *** (−6.66)	−0.129 *** (−6.45)	−0.116 *** (−5.72)
ln(UIG)	*β_UIG_*	−0.139 (−0.63)	−0.215 ** (−2.22)	−0.181 ** (−2.20)	−0.085 (−0.77)
ω × ln(PI)	θ *_PI_*				−0.096 ** (−2.42)
ω × ln(AGDP)	θ *_AGDP_*				0.448 *** (3.13)
ω × ln(UR)	θ *_UR_*				0.051 (0.13)
ω × ln(GER)	θ *_GER_*				0.049 (0.93)
ω × ln(UIG)	θ *_UIG_*				0.336 (1.42)
	λ		0.523 *** (7.10)		
	*ρ*			0.353 *** (5.74)	0.514 *** (7.08)

Note: ***, **, and * indicate significance at the 1%, 5%, and 10% levels, respectively.

**Table 6:** Estimation results of the SDM model under three weight matrices.

Variables	Coefficient	$\mathrm{Geographic}\;\mathrm{Matrix}\;(w_{ijd})$	$\mathrm{Economic}\;\mathrm{Matrix}\;(w_{ije})$	$\mathrm{Technology}\;\mathrm{Matrix}\;(w_{ijt})$
ln(PI)	*β_PI_*	0.008 (0.59)	0.003 (0.20)	0.016 (1.01)
ln(AGDP)	*β_AGDP_*	−0.854 *** (−17.46)	−0.862*** (−16.38)	−0.871 *** (−16.83)
ln(UR)	*β_UR_*	−0.081 (−0.52)	−0.346*** (−2.32)	−0.599 *** (−3.88)
ln(GER)	*β_GER_*	−0.116 *** (−5.72)	−0.129*** (−6.07)	−0.127 *** (−6.02)
ln(UIG)	*β_UIG_*	−0.085 (−0.77)	−0.144 (−1.44)	−0.186 * (−1.78)
ω × ln(PI)	θ *_PI_*	−0.096 ** (−2.42)	−0.055 (−1.05)	−0.125 ** (−2.42)
ω × ln(AGDP)	θ *_AGDP_*	0.448 *** (3.13)	−0.150 (−0.76)	−0.186 (−1.06)
ω × ln(UR)	θ *_UR_*	0.051 (0.13)	0.328 (0.79)	0.823 * (1.81)
ω × ln(GER)	θ *_GER_*	0.049 (0.93)	−0.080 (−1.31)	−0.133 ** (−2.81)
ω × ln(UIG)	θ *_UIG_*	0.336 (1.42)	−0.547 * (−1.91)	−0.437 (−1.52)
	ρ	0.514 *** (7.08)	0.365 *** (4.11)	0.200 ** (2.21)

Note: ***, **, and * indicate significance at the 1%, 5%, and 10% levels, respectively.

## Data Availability

The authors may provide raw data if necessary.
